# Tribolayer Architecture and Subsurface Crystallographic Evolution in Nanocrystalline Ni Under Distinct Sliding Conditions

**DOI:** 10.3390/ma19194063

**Published:** 2026-09-23

**Authors:** Wei Cai, Xiaobing Huang, Wanquan Zhu, Qiongyao He

**Affiliations:** 1 State Key Laboratory of Mechanical Transmission for Advanced Equipment, Chongqing University, Chongqing 400044, China; weicai@stu.cqu.edu.cn (W.C.); xiaobinghuang@cqu.edu.cn (X.H.); 2 International Joint Laboratory for Light Alloys, College of Materials Science and Engineering, Chongqing University, Chongqing 400044, China; 3 Laboratory for Ultrafast Transient Facility, Chongqing University, Chongqing 401331, China; 4 Southwest Technology and Engineering Research Institute, Chongqing 400039, China

**Keywords:** nanocrystalline metals, wear, crystallographic texture, correlation coefficient map, orientation mapping, four-dimensional scanning transmission electron microscopy

## Abstract

**Highlights:**

- Distinct sliding conditions produce different tribolayer architectures in nanocrystalline Ni coatings.
- Correlation coefficient maps enhance the visualization of worn subsurface features.
- 4D-STEM reveals shear-related texture in the worn subsurface.

**Abstract:**

Tribological loading can substantially modify the near-surface structures of nanocrystalline metals, yet the microstructural evolution of worn tribolayers remains insufficiently understood. Here, an electrodeposited nanocrystalline Ni coating with an average grain size of ~10 nm was investigated under ball-on-disk sliding against AISI 52100 steel in laboratory air at sliding speeds of 0.03 and 0.3 m/s with identical normal load and sliding distance. The worn surfaces and subsurface regions were characterized using laser confocal microscopy, transmission electron microscopy (TEM), energy-dispersive spectroscopy (EDS), and four-dimensional scanning transmission electron microscopy (4D-STEM). The steady-state coefficient of friction was higher at 0.03 m/s (~0.05) than at 0.3 m/s (~0.02). Low-speed sliding produced greater material pile-up and surface topographic changes, accompanied by the formation of an ~1 μm-thick porous oxide-rich tribolayer containing transferred Fe and isolated Ni-rich regions. In contrast, high-speed sliding generated a thinner (~300 nm) oxide layer above a ~150 nm metallic lamellar subsurface zone. 4D-STEM orientation analysis of reliably indexed near-surface regions further revealed local crystallographic orientation features consistent with $A_{1}^{*}$/$A_{2}^{*}$ and C components in the quasi-lamellar region at 0.03 m/s, whereas additional shear-related components, including C, B/$\bar{B}$, and $A_{1}^{*}$/$A_{2}^{*}$, were identified in the highly deformed subsurface region under the 0.3 m/s condition. Combined TEM observations and 4D-STEM crystallographic analysis demonstrate that different sliding conditions produce distinct tribolayer architectures, subsurface deformation structures, and crystallographic evolution in nanocrystalline Ni/AISI 52100 steel contacts.

## 1. Introduction

Nanocrystalline metallic coatings are widely studied because grain refinement can provide high strength, high hardness, and improved resistance to surface damage [1,2,3,4,5]. However, when these materials are subjected to sliding contact, the near-surface structure may evolve substantially from the as-deposited state. The microstructure controlling the subsequent response is therefore not necessarily the initial nanocrystalline microstructure, but rather the dynamically evolved tribolayer and subsurface region formed during sliding. In nanocrystalline Ni and Ni-based alloys [6,7], previous studies have reported sliding-induced grain growth [8,9], grain-boundary relaxation, mechanically mixed layers, and the formation of gradient or lamellar subsurface structures. These observations show that the wear of nanocrystalline metals is closely linked to microstructural evolution, and that the direct characterization of the worn surface and subsurface is essential for understanding their behavior.

Under ambient-air sliding, this structural evolution is further complicated by tribo-oxidation and counterbody material transfer. The tribolayer should be considered as a chemically and structurally heterogeneous product of the complete Ni/steel tribosystem, rather than as a simple deformed layer of the coating alone. Sliding speed can influence this evolution by changing the balance between mechanical shear, exposure time in air, oxidation, debris formation, and transfer-layer development [10,11,12]. However, how these coupled processes affect the architecture of the tribolayer and the crystallographic state of the underlying metallic subsurface remains insufficiently resolved. Conventional transmission electron microscopy (TEM) and selected-area electron diffraction (SAED) are powerful for identifying phases and general deformation structures, but they often average information over regions containing multiple grains and strong local orientation gradients. Conventional electron backscatter diffraction (EBSD) has provided important insight into wear-induced texture in coarse-grained face-centered cubic (FCC) metals, including the development of simple-shear texture components after sliding [13,14]. Direct crystallographic characterization of thin nanocrystalline tribolayers remains difficult [15] because the relevant structural features may be only a few tens to hundreds of nanometers thick.

In this context, four-dimensional scanning transmission electron microscopy (4D-STEM) [16,17] provides a particularly attractive approach because it records a diffraction pattern at every probe position, allowing structural and crystallographic information to be extracted from the same dataset. Recent developments in fast pixelated detectors [18] and automated pattern-matching workflows [19] have greatly expanded the capability of 4D-STEM for virtual diffraction imaging [20], orientation mapping, and the analysis of local crystallographic heterogeneity in nanostructured materials. In addition, correlation-coefficient mapping of neighboring diffraction patterns [21] can highlight structural discontinuities such as grain boundaries and local diffraction overlap, which is especially valuable in heterogeneous tribolayers.

Previous studies on this nanocrystalline Ni coating system have primarily focused on the consequences of sliding-induced structural modification, such as the influence of wear-induced surface changes on subsequent corrosion behavior [22]. However, the mechanisms by which different sliding histories reconstruct the tribolayer architecture, modify subsurface deformation structures, and reorganize crystallographic orientation at the nanoscale remain insufficiently understood. In particular, the relationship between the chemically evolved tribolayer and the underlying shear-induced crystallographic response has not been fully resolved.

Here, we investigate the dry sliding behavior of an electrodeposited nanocrystalline Ni coating with an average grain size of ~10 nm under two sliding speeds, 0.03 and 0.3 m/s, while keeping the normal load and total sliding distance constant. Surface morphology was characterized by laser confocal microscopy, and site-specific cross-sectional lamellae were examined by TEM, EDS, and 4D-STEM. Particular attention is given to the relation between sliding speed, tribolayer architecture, and local crystallographic evolution. The results show that both sliding conditions destabilize the initial nanocrystalline structure, but that they do so through distinctly different tribolayer evolution pathways.

## 2. Materials and Methods

### 2.1. Sample Preparation

Nanocrystalline Ni coatings were fabricated by direct-current electrodeposition on pure Cu substrates. A high-purity electrolytic Ni plate (99.997%) and a pure Cu plate (99.97%) were used as the anode and cathode, respectively. The electrodeposition electrolyte was a modified Watts-type nickel plating bath containing approximately 280 g/L NiSO_4_, 15 g/L NiCl_2_, and 35 g/L H_3_BO_3_ (Chongqing Chuandong Chemical (Group) Co., Ltd., Chongqing, China). Saccharin, sodium dodecyl sulfate, and 1,4-butynediol (Shanghai Aladdin Biochemical Technology Co., Ltd., Shanghai, China) were added at concentrations of approximately 1.0, 0.2, and 0.7 g/L, respectively, to promote grain refinement and improve coating quality. The electrolyte pH was adjusted to approximately 5 before deposition.

Electrodeposition was conducted in a 1 L glass beaker placed in a thermostatic water bath. The bath temperature was maintained at approximately 50 °C, and the distance between the facing anode and cathode surfaces was kept at approximately 8 cm. A magnetic stirrer (Gongyi Yuhua Instrument Co., Ltd., Gongyi, China) was operated at approximately 400 rpm to maintain homogeneous mass transport during deposition. A direct-current power supply (Shenzhen LODESTAR Precision Tools Co., Ltd., Shenzhen, China) was operated under galvanostatic conditions with a current density of 2 A/dm^2^ for approximately 4 h. The resulting Ni coatings were dense and fully nanocrystalline, with a thickness of approximately 60 μm.

### 2.2. Tribological Test

Dry sliding wear tests were performed in a ball-on-disk configuration using a MFT-5000 multifunction tribometer (Rtec Instruments, San Jose, CA, USA). The tests were conducted in laboratory air at 20 °C and 40% relative humidity. The nanocrystalline Ni specimens were fixed in a self-centering chuck and rotated against a stationary AISI 52100 bearing-steel ball with a diameter of 9.524 mm and a hardness of 63 HRC. A constant normal load of 5 N was applied in all tests. Two sliding speeds were selected, 0.03 and 0.3 m/s, while the total sliding distance was kept constant so that the extent of wear and subsurface evolution could be compared directly. The experimental configuration is schematically illustrated in Figure 1. The detailed test parameters are summarized in Table 1. The friction and surface-profile measurements were used here to provide tribological context for the detailed microstructural characterization. Because the present study focused on site-specific structural and crystallographic evolution, the results should be interpreted for the present nanocrystalline Ni/AISI 52100 steel sliding pair and test conditions. Due to the limited availability of the nanocrystalline Ni coating specimens, the low-speed and high-speed sliding tests were performed on different track radii of the same specimen. This approach minimized the influence of specimen-to-specimen variations on the comparison between the two sliding conditions. The obtained friction and wear results correspond to the investigated sliding conditions, and additional repeated measurements using independent specimens were not performed in this study. The two sliding speeds (0.03 and 0.3 m/s) were selected as representative low and high speed conditions differing by one order of magnitude. The purpose of selecting these two conditions was to investigate the influence of distinct sliding histories on friction-induced microstructural and crystallographic evolution rather than to establish a continuous sliding-speed dependence. The selected conditions provide sufficiently different contact frequencies, exposure histories, and deformation/oxidation environments for a comparison of tribolayer evolution and subsurface structural changes.

### 2.3. Macroscopic and Subsurface Characterization

The worn surface morphology was examined using laser confocal microscopy with an LSM900 microscope (ZEISS, Oberkochen, Germany). The reference plane for wear-depth measurement was determined by fitting a least-squares plane to the unworn regions surrounding the wear track. The fitted plane was defined as z=0, and the height variation of the worn region was evaluated relative to this reference plane. Cross-sectional lamellae were prepared by focused ion beam (FIB) lift-out using a Helios G4 UX system (Thermo Fisher Scientific, Hillsboro, OR, USA). Bright-field (BF) TEM, dark-field (DF) TEM, selected-area electron diffraction, and high-resolution transmission electron microscopy (HRTEM) imaging were performed. Conventional TEM and SAED analyses were conducted using a JEM-2100 transmission electron microscope (JEOL Ltd., Tokyo, Japan) operated at 200 kV. HRTEM and chemical analysis were carried out using the JEM-F200 equipped with an Oxford Xplore EDS system (Oxford Instruments, Abingdon, UK).

### 2.4. 4D-STEM Data Acquisition and Processing

4D-STEM was performed using a Spectra 200 Cs-corrected STEM (Thermo Fisher Scientific, Hillsboro, OR, USA) equipped with an electron microscope pixel array detector (EMPAD, 128 × 128 pixels; Thermo Fisher Scientific, Hillsboro, OR, USA). The exposure time for each diffraction pattern was 5 ms, and the probe size was approximately 1.5 nm. For each specimen, two datasets were acquired, with scan sizes of 512 × 512 and 512 × 200 pixels and corresponding step sizes of 1.1 and 2.1 nm, respectively. To visualize local crystallographic discontinuities, neighborhood diffraction pattern correlation coefficient maps were calculated from the similarity of diffraction patterns at adjacent probe positions. The general concept is related to the correlation-coefficient mapping approach reported in Ref. [21], but the implementation used here was modified for the present 4D-STEM datasets. The diffraction patterns were first aligned to correct possible pattern drift during acquisition. A background pattern was then estimated as the pixel-wise minimum intensity over all diffraction patterns in the dataset and subtracted from each individual pattern. After background subtraction, each diffraction pattern was treated as a vector, $I_{i}\left(q\right)$, where *i* denotes the probe position and **q** denotes the detector pixel coordinate. The similarity between neighboring diffraction patterns was calculated using the cosine similarity ($C_{ij}$):

$$C_{ij}=\frac{\sum_{q}I_{i}\left(q\right)I_{j}\left(q\right)}{\sqrt{\sum_{q}{I_{i}\left(q\right)}^{2}}\sqrt{\sum_{q}{I_{j}\left(q\right)}^{2}}}$$

where *j* denotes an adjacent probe position. For each probe position *i*, the neighborhood correlation coefficient (${\bar{C}}_{i}\;$) was obtained by averaging the similarity values over all valid neighboring positions:

$${\bar{C}}_{i}=\frac{1}{N_{i}}\sum_{j\in N\left(i\right)}C_{ij}$$

where $N\left(i\right)$ is the set of valid adjacent probe positions and $N_{i}$ is the corresponding number of neighbors. Thus, $N_{i}$ = 8 for interior probe positions, $N_{i}$ = 5 for edge positions, and $N_{i}$ = 3 for corner positions. The resulting two-dimensional map highlights local changes in diffraction information. Lower correlation values appear darker and indicate regions where neighboring diffraction patterns differ, which may correspond to grain boundaries, low-angle boundaries, intragranular misorientation, defects, or diffraction overlap.

In addition, diffraction patterns from user-defined regions were summed to produce virtual selected-area diffraction patterns for selected microstructural domains using in-house codes. Crystallographic orientation mapping was carried out using the py4DSTEM package (version 0.14.9) by matching the experimental diffraction patterns to simulated pattern libraries [23]. The reliability of each indexing solution was evaluated based on the correlation coefficients obtained from the template-matching procedure. Following the reliability assessment approach for automated crystal orientation mapping, the reliability factor (R) was calculated as [24]:

$$R=100\left(1-Q_{2}/Q_{1}\right)$$

where $Q_{1}$ and $Q_{2}$ represent the correlation coefficients of the first and second highest matching diffraction templates, respectively. The reliability factor describes the uniqueness of the indexing solution; a larger value indicates a greater difference between the best and alternative solutions and therefore higher confidence in the assigned orientation. Previous template-matching-based orientation mapping studies have shown that reliability values above approximately 15 generally correspond to reliable indexing solutions, whereas lower values may arise from ambiguous diffraction solutions associated with overlapping crystals or other sources of indexing uncertainty [24]. Therefore, reliability maps were used to evaluate the confidence of the orientation mapping and to identify regions where diffraction overlap could influence the indexing results. The orientation distribution function (ODF) was calculated using AztecCrystal software (version 2.1) based on the indexed orientation datasets. Only regions with sufficiently high indexing reliability were considered for subsequent crystallographic texture analysis, while regions exhibiting significantly reduced reliability were treated with caution because of the possible influence of diffraction overlap effects.

Grain-size statistics were obtained through a multi-step grain identification and segmentation procedure. First, individual grains within the analyzed subsurface regions were identified by combining conventional dark-field TEM images, grain-boundary information reconstructed from the 4D-STEM correlation coefficient maps, and the corresponding virtual dark-field images. These complementary datasets were used to distinguish adjacent grains and determine the locations of grain boundaries, particularly in regions where diffraction contrast alone was insufficient for unambiguous grain separation. After identifying the grain boundaries, individual grains were segmented from the analyzed regions, and the projected area of each separated grain was measured. Because the deformation-induced subsurface grains exhibited irregular and elongated morphologies rather than ideal equiaxed shapes, the grain size was quantified using the equivalent circular diameter based on the measured projected grain area. The equivalent circular diameter ($d_{eq}$) was calculated according to:

$$d_{eq}=2\sqrt{\frac{A}{\pi }}$$

where $d_{eq}$ is the equivalent circular diameter and A represents the projected area of an individual grain.

## 3. Results

### 3.1. Structural Characterization of the As-Deposited State

Plan-view BF TEM images of the as-deposited coating (Figure 2a) revealed a dense and uniform nanocrystalline structure with predominantly equiaxed grains. Statistical analysis indicates an average grain size of approximately 10 nm. The corresponding plan-view DF image and SAED pattern (Figure 2b,c) showed continuous Debye–Scherrer rings with nearly uniform azimuthal intensity, indicating the absence of a pronounced in-plane preferred orientation.

In contrast, the cross-sectional microstructure was slightly anisotropic (Figure 2d,e). Cross-sectional BF and DF TEM images showed grains that were weakly elongated along ND, consistent with the growth direction during electrodeposition. The major axis of some grains extended to several tens of nanometers. The cross-sectional SAED pattern (Figure 2f) differed from the plan-view pattern by showing non-uniform intensity along the diffraction rings, especially the first ring highlighted in Figure 2f. This azimuthal intensity variation indicates that the as-deposited coating possesses a {1 1 1} fiber texture aligned approximately with ND. A weaker intensity variation was also observed for the {2 0 0} ring, suggesting the presence of a minor {1 0 0} fiber texture along ND in the as-deposited state.

### 3.2. Friction and Wear Behavior

The coefficient of friction (COF) was continuously recorded as a function of sliding distance for both sliding speeds (Figure 3). In both cases, the friction curves displayed three characteristic stages: an initial sharp increase in COF at the onset of sliding, followed by a gradual decrease accompanied by noticeable fluctuations, and finally a relatively stable steady-state regime that persisted for the remainder of the test. This evolution is consistent with the classical break-in, running-in, and steady-state stages of the wear process [5,25].

For both the 0.03 and 0.3 m/s tests, the transition to the steady-state regime occurred after a sliding distance of approximately 50 m. Because the total distance was the same but the speed differed by one order of magnitude, the elapsed time required to reach this transition was very different: about 28 min for 0.03 m/s and about 2.8 min for 0.3 m/s. The steady-state COF at 0.03 m/s was approximately 0.05, which was more than twice that measured at 0.3 m/s (~0.02).

To quantify the corresponding surface deformation, 3D confocal surface profiles were acquired from the wear tracks (Figure 4). For both conditions, the track width was approximately 600 μm. Using the adjacent unworn surface as the zero-height reference, the morphology of material removal and material pile-up could be compared directly. The wear track formed at 0.03 m/s was dominated by severe material pile-up. The 3D topography (Figure 4a) did not show a clear and continuous central valley; instead, the track was characterized by large and continuous positive-height features. The highest asperities reached approximately 80 μm above the reference plane. The corresponding 2D profiles (Figure 4b) showed only limited and isolated regions below the original surface level, indicating that material redistribution by pile-up is the dominant topographic feature under this condition. In contrast, the wear track formed at 0.3 m/s shown in Figure 4c,d contained a clear central valley associated with material removal and local compaction. This valley was bordered by positive-height ridges generated by lateral extrusion and pile-up. Local asperities exceeded 70 μm above the reference plane.

The qualitative observations were corroborated by quantitative volumetric analysis (Table 2). The two sliding conditions were compared under the same normal load and total sliding distance; however, differences in sliding velocity were accompanied by changes in testing duration, number of revolutions, and track radius. Therefore, the observed differences represent the response of the coating under two specific sliding conditions rather than an isolated effect of sliding velocity. Under these conditions, high-speed sliding resulted in greater downward material removal, giving a valley volume of 2.60 × 10^5^ μm^3^; the corresponding value for slow-speed sliding was 6.01 × 10^4^ μm^3^. Conversely, slow-speed sliding caused markedly greater upward material pile-up, with a pile-up volume of 1.97 × 10^6^ μm^3^ compared with 7.70 × 10^5^ μm^3^ for high-speed sliding. As a result, the total volumetric displacement, defined as the absolute sum of all topographical deviations from the unworn surface, reaches 2.03 × 10^6^ μm^3^ under slow-speed sliding, which was approximately twice the value measured for high-speed sliding (1.03 × 10^6^ μm^3^). This result is consistent with the higher steady-state COF observed at the lower sliding velocity.

### 3.3. Microstructure and Chemistry of the Tribolayers

Cross-sectional TEM observations of the specimen tested at 0.03 m/s (Figure 5a) revealed a two-layer architecture. The uppermost region was a porous tribolayer with a thickness of approximately 1 μm. EDS mapping (Figure 5b) showed strong oxygen enrichment across this layer, together with a clear Fe signal throughout the tribolayer. This indicates extensive material transfer from the AISI 52100 steel counterbody and the formation of an oxide-rich mechanically mixed layer. In addition to the transferred Fe-containing material, isolated Ni-rich domains marked by red arrows were present within the tribolayer, suggesting that metallic Ni fragments are entrained in the oxidized surface layer during sliding.

The SAED pattern acquired from the tribolayer (Figure 5c) displayed continuous diffraction rings, indicating a nanocrystalline tribolayer without a pronounced crystallographic texture. As summarized in Table 3, the measured diffraction-ring spacings showed good agreement with the major reflections of NiO. Considering the Fe transfer from the steel counterbody detected by EDS, the possible contribution of Fe-containing oxide phases was further evaluated. FeO possesses the same FCC rocksalt structure as NiO and a relatively close lattice parameter (NiO: a = 0.4177 nm; FeO: a = 0.4326 nm), resulting in partial overlap between their diffraction reflections. Therefore, the contribution of FeO-type oxide cannot be completely excluded based solely on SAED analysis. In contrast, the measured diffraction spacings showed no clear agreement with the characteristic reflections of Fe_2_O_3_ or Fe_3_O_4_. Together with the presence of metallic Ni fragments observed within the tribolayer (Figure 5a), these results suggest that the tribolayer consists of a NiO-dominated mixed oxide phase with possible Fe-containing oxide contributions and mechanically entrained metallic fragments generated during sliding. High-magnification bright- and dark-field TEM images (Figure 5e,f) further showed that the nanocrystalline grains within this mixed tribolayer were approximately 3–20 nm in size.

The microstructure formed at 0.3 m/s can be divided into three distinct regions (Figure 6a). The outermost layer was again oxide-rich, but it was much thinner, with a thickness of only ~300 nm. Unlike the corresponding layer in the low-speed sample, this outermost layer was dense rather than porous. EDS analysis (Figure 6b) confirmed oxygen enrichment and the presence of Fe-containing transferred material, indicating that both tribo-oxidation and counterbody transfer remain active under both sliding conditions. The diffraction pattern from the upper layer (Figure 6c) exhibited diffraction features similar to those observed in the low-speed tribolayer, including reflections associated with the NiO-type oxide structure and metallic Ni. Together with the EDS results showing Fe-containing transferred material, these observations indicate that the oxide-rich layer formed under both sliding conditions had a similar mixed character, consisting mainly of NiO-type oxide with possible Fe-containing oxide contributions and mechanically entrained metallic fragments.

Higher-magnification BF- and DF-TEM images (Figure 6e,f) revealed that beneath the thin oxide layer, the specimen tested at 0.3 m/s contained a nanolamellar subsurface region approximately 150 nm thick. The individual lamellae were typically ~50 nm in thickness. Such a highly aligned morphology is characteristic of severe interfacial shear deformation, in which the crystalline matrix is stretched and flattened parallel to the sliding direction.

### 3.4. 4D-STEM Observations

To further resolve the crystallographic heterogeneity of the worn subsurface, the 4D-STEM datasets were analyzed using correlation coefficient maps. For the specimen tested at 0.03 m/s, the correlation map revealed a complex near-surface architecture (Figure 7a). Beneath the oxide layer (Zone 1), an approximately 70 nm-thick quasi-lamellar region (Zone 2) could be resolved, although it was not clearly apparent in the conventional BF- and DF-TEM images (Figure 5e,f). Grain-size statistics were obtained by identifying individual grains from the combined information of conventional dark-field TEM images, grain-boundary networks reconstructed from the 4D-STEM correlation coefficient maps (Figure 6a and Figure 7a), and the corresponding virtual dark-field images. After identifying and segmenting individual grains, the projected area of each grain was measured. The equivalent grain diameter was calculated from the projected grain area using the equivalent-circle method. For the low-speed specimen, 92 grains in Zone 2 and 178 grains in Zone 3 were analyzed. For the high-speed specimen, 96 grains in Zone 2 and 166 grains in Zone 3 were analyzed.

The grains in Zone 2 were elongated, with their major axes approximately parallel to the sliding direction. The equivalent circle diameter distribution of grains in Zone 2 was relatively broad, with a mean grain size of 38 ± 19 nm (Figure 7f). The correlation map further showed that some coarsened grains exhibited intragranular diffraction variations, indicating that they were not crystallographically uniform. The summed diffraction pattern from Zone 2 (Figure 7b) contained discrete reflections characteristic of coarsened metallic Ni grains while still retaining a preferential distribution of the {1 1 1} and {2 0 0} reflections along ND. A representative diffraction pattern from a quasi-lamellar grain in Zone 2 (Figure 7d) is consistent with a single orientation and can therefore be indexed. The elongation of the higher-order reflections suggests local lattice rotation or intragranular misorientation.

In Zone 3, several coarsened subsurface grains were clearly resolved, with some exhibiting morphological dimensions exceeding ~100 nm. Compared with Zone 2, the equivalent circle diameter distribution of grains in Zone 3 was dominated by much finer grains, with a mean value of 11 ± 6 nm (Figure 7g). It should be noted that the grain sizes obtained from statistical analysis represent equivalent circular diameters rather than the maximum dimensions directly observed in the TEM images. Because the subsurface grains generated during sliding deformation often exhibit elongated or irregular morphologies, the longest grain axis observed in TEM images does not necessarily correspond to the equivalent circular diameter calculated from the projected area. For example, elongated grains with morphological dimensions exceeding 100 nm may still exhibit smaller equivalent circular diameters depending on their projected area and aspect ratio. Therefore, the absence of a significant fraction of grains above 100 nm in the statistical distribution does not indicate the absence of locally elongated or coarsened structures, but reflects the difference between morphological dimensions and area-based statistical measurements.

These grains do not simply retain the original columnar morphology of the coating. Instead, some elongated grains were inclined away from ND toward SD, suggesting reorientation under sliding-induced shear. The summed diffraction pattern from Zone 3 (Figure 7c) broadly resembled that of the as-deposited state. Nevertheless, the microstructure in this region was heterogeneous, and grain overlap was common. Consequently, many local diffraction patterns contained contributions from multiple grains along the beam path (Figure 7e), preventing reliable indexing of individual grain orientations.

At 0.3 m/s, the specimen also showed substantial crystallographic complexity in the neighborhood correlation coefficient map (Figure 8a). Beneath Zone 1, weak dark traces were observed within the grains in the lamellar region; these were attributed to low-angle grain boundaries and suggest that dislocation-mediated deformation is active in this region. The summed diffraction pattern from Zone 2 (Figure 8b) retained discrete reflections characteristic of coarsened grains, but exhibited weaker intensity along the {1 1 1} ring in ND. At the same time, the {2 2 0} reflections displayed a stronger preferential distribution along SD than in the as-deposited state. A representative diffraction pattern from Zone 2 (Figure 8d) also showed slight elongation of the reflections, indicating local lattice distortion and/or defect accumulation. In contrast, the summed diffraction pattern from Zone 3 (Figure 8c) was broadly similar to that of the as-deposited state. Nevertheless, a typical local diffraction pattern from Zone 3, taken from the region marked by the green cross in Figure 8a, again indicated overlap of diffraction contributions from more than one grain along the beam direction (Figure 8e). The equivalent circle diameter distributions of grains of Zones 2 and 3 are summarized in Figure 8f and g, respectively. Compared with the specimen tested at 0.03 m/s (Figure 7f,g), the average grain sizes remained nearly unchanged, with mean values of 34 ± 16 nm in Zone 2 and 10 ± 6 nm in Zone 3.

4D-STEM datasets were indexed to characterize the crystallographic orientations of the near-surface deformation structures (Figure 9a,b). Because substantial grain overlap in Zone 3 introduced greater uncertainty in orientation indexing, the orientation analysis was focused primarily on Zone 2, where grain overlap was less pronounced. The IPF orientation maps in Figure 9a,b showed the regions with successfully indexed crystallographic orientations, corresponding to the near-surface lamellar structures in Zone 2. The colors represent the crystallographic direction parallel to the sliding direction, while the superimposed grayscale contrast reflects the confidence of local orientation indexing. The corresponding reliability maps (Figure 9c,d) further confirmed that the indexed regions used for orientation analysis exhibited relatively high indexing reliability, whereas regions with reduced reliability were mainly associated with complex diffraction overlap or ambiguous diffraction signals. The specimen tested at 0.03 m/s (Figure 9a) exhibited a quasi-lamellar structure containing elongated grains together with some nearly equiaxed grains. In contrast, the specimen tested at 0.3 m/s (Figure 9b) showed a more clearly developed lamellar structure, with elongated grains extending predominantly along the sliding direction. The relatively high reliability of the indexed near-surface grains indicates that these orientation variations can be reliably resolved despite the nanoscale deformation and partial diffraction overlap.

Because the formation of these lamellar structures is associated with severe plastic deformation during sliding, the crystallographic orientation variation within individual grains was further analyzed to evaluate the deformation-induced orientation heterogeneity. Misorientation-angle profiles were therefore extracted along the white dashed lines within representative grains in Figure 9a,b. For each profile, the misorientation angle at each position was calculated relative to the crystallographic orientation at the corresponding starting point (point M in Figure 9a and point N in Figure 9b). As shown in Figure 9e,f, appreciable variations in misorientation angle occurred within individual near-surface grains in both specimens, demonstrating the presence of intragranular orientation gradients. These orientation gradients indicate non-uniform crystallographic deformation within the near-surface grains and may be associated with deformation-induced defects, such as dislocation accumulation, during repeated tribological loading.

Based on the 4D-STEM orientation characterization, the crystallographic textures of the near-surface Zone 2 regions with reliable orientation indexing in the specimens tested under the two sliding conditions were further analyzed using orientation distribution functions (ODFs). The ODFs were evaluated using the φ_2_ = 45° sections shown in Figure 10, with the ideal FCC shear texture components presented in Figure 10a as a reference [26]. For the specimen tested at 0.03 m/s, the ODF of the quasi-lamellar region (Figure 10b) was characterized primarily by the $A_{1}^{*}$ ({1 −1 1}<1 1 2>), $A_{2}^{*}$ ({1 1 −1}<2 −1 1>), and C ({0 0 1}<1 1 0>) components. In comparison, the lamellar region in the specimen tested at 0.3 m/s (Figure 10c) exhibited the same $A_{1}^{*}$/$A_{2}^{*}$ and C components, together with additional B ({1 −1 2}<1 1 0>) and $\bar{B}$ ({−1 1 −2}<−1 −1 0>) components. These results indicate that characteristic FCC shear texture components were present in Zone 2 under both sliding conditions, whereas the specimen tested at 0.3 m/s exhibited a broader range of shear-texture components. Similar shear texture components have been reported in the near-surface regions of surface-treated Al alloys [27,28] and in metals such as Cu [13] and Ni [29,30].

To further examine the deformation structures associated with the shear-related texture evolution, additional high-resolution transmission electron microscopy (HRTEM) and crystallographic analyses were performed on the subsurface lamellar region of the specimen tested at 0.3 m/s. As shown in Figure 11a, clear twin boundaries (TBs) were observed within the highly deformed lamellar structure, indicating the occurrence of deformation twinning during sliding-induced plastic deformation. The corresponding fast Fourier transform (FFT) pattern (Figure 11b) can be indexed based on the FCC Ni structure along the [011] zone axis. The diffraction spots from the matrix and twin regions exhibited a characteristic twin-related crystallographic relationship, further supporting the identification of deformation twins. These observations provide direct microstructural evidence that twinning occurs within the severely deformed subsurface region during sliding. However, the relative contribution of twinning compared with other deformation processes cannot be quantitatively determined based on the present observations alone.

## 4. Discussion

### 4.1. Wear Mechanisms Under Ambient-Air Dry Sliding

The combined friction, confocal, TEM, EDS, and 4D-STEM results show that changing the sliding speed changes the dominant wear pathway even when the total sliding distance is fixed. The low-speed condition, 0.03 m/s, produced a higher steady-state coefficient of friction, much greater material pile-up, and a thick porous tribolayer containing oxygen, transferred Fe, and residual Ni. In contrast, the high-speed condition, 0.3 m/s, produced a lower steady-state friction coefficient, a much thinner oxide-rich surface layer, and a more distinct metallic lamellar subsurface region. Because the confocal analysis captures both materials removed below the original surface and material displaced above it, the slow-speed condition is best described as causing greater total topographic displacement rather than simply greater net material loss.

This trend differs from the inert-atmosphere behavior reported by Padilla et al. [12] for nanocrystalline Ni-Fe, where low sliding speeds favored low friction and higher sliding speeds promoted high friction through tribolayer instability and delamination. The contrast is important. Padilla and related work deliberately suppressed tribo-oxidation by testing in dry nitrogen, allowing the friction response to be interpreted primarily in terms of mechanical tribolayer stability. In the present study, the tests were performed in ambient air, and both EDS and diffraction showed extensive oxygen enrichment together with Fe transfer from the counterbody. The opposite speed-friction trend therefore suggests that in the present system, tribo-oxidation and third-body evolution play a much larger role than in the inert Ni-Fe studies.

Another important point is that the steady-state friction coefficients measured here are substantially lower than those commonly reported for nanocrystalline Ni and related Ni-based alloys under dry sliding. Previous studies on nanocrystalline Ni [10,11,31] and nanocrystalline Ni-Fe [12] typically reported steady-state values in the range of ~0.2–0.8, depending on contact stress, sliding speed, environment, and tribosystem. The present values therefore suggest that the friction response is strongly influenced by the specific interfacial chemistry and third-body evolution that occur in the current ambient-air Ni/steel contact. In particular, the EDS and diffraction results showed that both sliding conditions generate an oxide-bearing tribolayer containing transferred material from the steel counterbody, so the contact cannot be regarded as a simple metallic Ni/steel interface throughout the test.

For the 0.03 m/s condition, the same sliding distance corresponded to an exposure time approximately ten times longer than that at 0.3 m/s. That longer exposure in air likely promotes repeated adhesive transfer from the steel ball, oxidation of entrained material, and the accumulation of a porous mechanically mixed oxide layer [32]. Earlier work on nanocrystalline Ni in ambient air likewise showed that oxidized debris can agglomerate into a compacted protective tribolayer and reduce friction relative to inert conditions [10]. In the present case, however, the low-speed tribolayer was thicker, chemically heterogeneous, and porous, indicating repeated reworking during sliding. The large pile-up and the absence of a well-defined central valley are therefore consistent with a third body that is repeatedly compacted, fractured, and extruded.

Under the 0.3 m/s condition, oxidation and transfer still occurred, but they remained confined to a much thinner surface layer. The dominant subsurface feature was instead the metallic lamellar zone beneath the oxide scale. This architecture suggests that a larger fraction of the imposed strain is accommodated within a metallic shear zone rather than within a thick mechanically mixed oxide layer. Such a more organized near-surface deformation structure is consistent with the lower steady-state friction and the smaller total topographic displacement observed at high speed.

Because the two sliding tests were performed at different track radii (5 and 10 mm), the possible influence of the initial contact condition on tribolayer evolution was evaluated. The two tests employed the same AISI 52100 steel ball, identical normal load, and the same Ni coating/counterbody material pair. Therefore, the nominal Hertzian contact condition was expected to be identical for both sliding conditions. The Hertzian contact radius (a) was calculated according to classical elastic contact theory [33]:

$$a={\left(\frac{3F_{N}R}{4E^{*}}\right)}^{1/3}$$

where $F_{N}$ is the normal load, R is the radius of the steel ball, and $E^{*}$ is the reduced elastic modulus. The reduced elastic modulus was calculated using:

$$\frac{1}{E^{*}}=\frac{1-\nu_{1}^{2}}{E_{1}}+\frac{1-\nu_{2}^{2}}{E_{2}}$$

where $E_{1}\;$ and $\nu_{1}\;$ correspond to the Young’s modulus and Poisson’s ratio of the AISI 52100 steel ball, while $E_{2}\;$ and $\nu_{2}\;$ represent those of the nanocrystalline Ni coating. Using $E_{1}=210\;$ GPa and $\nu_{1}=0.30\;$ for AISI 52100 steel and $E_{2}=200\;$ GPa and $\nu_{2}=0.31\;$ for Ni [34], the reduced elastic modulus was calculated to be approximately 113 GPa. With a ball diameter of 9.524 mm and a normal load of 5 N, the Hertzian contact radius was calculated to be approximately 54.2 μm.

The corresponding maximum Hertzian contact pressure ($p_{0}$) [35] was calculated according to:

$$p_{0}=\frac{3F_{N}}{2\pi a^{2}}$$

where a  is the Hertzian contact radius. The calculated maximum contact pressure was approximately 810 MPa. Because the same counterbody geometry, normal load, and material pair were used for both sliding conditions, the Hertzian contact radius and maximum contact pressure were identical. Therefore, the distinct tribolayer architectures observed under different sliding speeds cannot be attributed to differences in the initial nominal contact mechanics, but rather to different sliding histories associated with velocity and exposure duration.

The markedly different oxidation behavior at the two sliding speeds should also be considered in terms of both the rate and duration of frictional energy input. The instantaneous frictional power can be estimated as $P_{f}=\mu F_{N}v$. Using the steady-state COFs of approximately 0.05 and 0.02, the corresponding frictional powers were approximately 7.5 mW at 0.03 m/s and 30 mW at 0.3 m/s, respectively. Thus, the higher sliding speed produces a larger instantaneous rate of frictional energy dissipation despite its lower COF. However, because both tests were conducted over the same sliding distance of 300 m, the lower-speed test lasted ten times longer (10,000 versus 1000 s) and dissipated a larger total frictional work, approximately 75 J compared with 30 J at 0.3 m/s. Taking the measured wear-track width of approximately 600 μm and the respective track radii of 5 and 10 mm into account, the cumulative frictional work normalized by the nominal wear-track area was approximately 4.0 and 0.8 MJ m^−2^ at 0.03 and 0.3 m/s, respectively. Therefore, although higher sliding speed may favor larger transient heat generation and potentially higher local flash temperatures at individual asperity contacts, the low-speed condition subjects the wear track to a substantially longer period of repeated mechanical interaction and a greater cumulative frictional-energy input per unit nominal track area. This distinction is important for interpreting the thicker oxide-rich tribolayer formed at 0.03 m/s. Rather than implying that the low-speed surface necessarily experiences a higher instantaneous temperature, the pronounced oxidation is more reasonably associated with the longer tribological exposure, repeated generation and incorporation of fresh metallic surfaces and wear debris, and continued oxidation and mechanical reworking of the third-body layer. Local frictional heating may accelerate these processes, but the present results do not require a high track-averaged temperature to account for the observed oxide-rich tribolayer.

To further evaluate the possible contribution of frictional heating to the different tribolayer evolution behaviors, the thermal transport regime during sliding was assessed using the Péclet number. For a moving heat source in a sliding contact, the Péclet number (Pe) is defined as [36]:

$$Pe=\frac{va}{2\alpha }$$

where v  is the sliding velocity, a  is the characteristic contact radius, and α  is the thermal diffusivity of the material. The Péclet number represents the competition between heat transport caused by the movement of the frictional heat source and heat diffusion into the bulk material. A larger value indicates a greater tendency for transient heat accumulation during sliding [37].

The thermal diffusivity (α) of nanocrystalline Ni was calculated using:

$$\alpha =\frac{k}{\rho C_{p}}$$

where k, ρ, and $C_{p}$ are the thermal conductivity, density, and specific heat capacity, respectively. Using the reported thermal properties of nanocrystalline Ni (k=67 W m^−1^ K^−1^, ρ=8900 kg m^−3^, and $C_{p}=440\;J$ kg^−1^ K^−1^) [38,39,40], the thermal diffusivity was calculated as:

$$\alpha =\frac{67}{8900\times 440}=1.71\times 10^{-5}\;m^{2}\;s^{-1}$$

Accordingly, the Péclet numbers for the two sliding conditions were calculated as:

$$Pe_{0.03}=\frac{0.03\times 54.2\times 10^{-6}}{2\times 1.71\times 10^{-5}}=0.047$$

and

$$Pe_{0.3}=\frac{0.3\times 54.2\times 10^{-6}}{2\times 1.71\times 10^{-5}}=0.475$$

The calculated Péclet numbers were below unity for both sliding conditions, indicating that heat diffusion remained significant during sliding and that neither condition corresponded to a strongly heat-accumulating thermal regime. Nevertheless, the approximately tenfold increase in Péclet number at the higher sliding speed indicates an increased tendency for transient thermal accumulation. Therefore, although the higher-speed condition generates a larger instantaneous frictional power, the different tribolayer structures cannot be attributed solely to instantaneous temperature effects. Instead, the thicker oxide-rich tribolayer formed under low-speed sliding is more reasonably related to the longer exposure time, repeated oxidation of newly generated metallic surfaces, and continuous mechanical reworking of third-body debris.

Both sliding conditions modified the initial ~10 nm nanocrystalline structure, with increased grain sizes observed in localized subsurface regions. However, the expanded grain-size statistics indicate that this structural evolution is heterogeneous rather than a uniform grain-growth process throughout the coating. The grain-size distributions were obtained after individual grain identification and segmentation using the combined information from conventional dark-field TEM images, 4D-STEM correlation coefficient maps, and virtual dark-field images. The grain size was then quantified using the equivalent circular diameter calculated from the projected area of each segmented grain. This area-based parameter was selected because the deformation-induced subsurface grains do not necessarily maintain equiaxed morphologies and often exhibit elongated or irregular shapes after sliding deformation. Compared with the maximum grain dimension, the equivalent circular diameter provides a more consistent statistical measure that is less sensitive to grain elongation and observation orientation.

The presence of locally modified grain structures away from the immediate sliding interface suggests that friction-induced deformation and recovery processes extend into the subsurface region. Because the magnitude of stress and thermal contribution is expected to decrease with depth, the observed subsurface structural variation likely reflects differences in local deformation history, stored deformation energy, and microstructural stability within the electrodeposited coating. Previous studies on nanocrystalline Ni-W have demonstrated that sliding can induce heterogeneous subsurface structural evolution, including grain-size modification and gradient microstructure formation, through repeated mechanical deformation and dynamic rearrangement of the near-surface region [41]. Similar deformation-induced structural rearrangements have also been reported in atomistic simulations of nanocrystalline metals, where severe local deformation promotes grain-boundary-mediated structural evolution. Therefore, the grain-size variations observed in the present study are interpreted as evidence of heterogeneous subsurface structural evolution rather than direct evidence of a specific grain-growth mechanism.

### 4.2. Effects of Sliding Conditions on Tribolayer Architecture and Texture Development

The differences between the two sliding conditions are reflected not only in the tribolayer thickness but also in the nature of the near-surface architecture and the development of shear-induced crystallographic features. At 0.03 m/s, the near-surface region was dominated by a thick oxide-rich mechanically mixed tribolayer, beneath which only a thin quasi-lamellar metallic region was detected. In contrast, at 0.3 m/s, the oxide-rich surface layer was considerably thinner, and a more clearly developed ~150 nm metallic lamellar zone formed beneath the tribolayer. These observations suggest that the two sliding conditions accommodate the imposed deformation through different pathways. Under the low-speed condition, the interfacial deformation is strongly coupled with oxidation, material transfer, and mechanical mixing within a heterogeneous tribolayer, whereas under the high-speed condition, a larger fraction of the imposed shear deformation is accommodated within the metallic subsurface region.

It should be noted that the two sliding conditions investigated in this study involve coupled changes in sliding velocity, exposure history, number of repeated contacts, and track geometry. Therefore, the observed differences in tribolayer architecture should not be attributed exclusively to sliding velocity. Nevertheless, sliding velocity itself may contribute to the different deformation responses because nanocrystalline Ni exhibits strain-rate-sensitive plastic deformation behavior. Schwaiger et al. demonstrated that nanocrystalline Ni exhibits pronounced strain-rate sensitivity, indicating that its plastic deformation response depends strongly on the imposed deformation rate [42]. More directly, Shafiei and Alpas reported that the friction and wear behavior of nanocrystalline Ni changed significantly with increasing sliding speed even under an argon atmosphere, where oxidation effects were minimized, and attributed this behavior to the rate-dependent near-surface deformation response of nanocrystalline Ni [43]. Because nanocrystalline Ni exhibits strain-rate-sensitive deformation behavior, different sliding velocities may therefore produce different surface and subsurface deformation responses. In the present study, this rate-dependent deformation behavior may contribute to the more clearly developed metallic lamellar region observed at 0.3 m/s.

In addition to the mechanical effect of sliding velocity, the exposure history between successive sliding contacts can significantly influence tribochemical evolution. During circular sliding, a specific position on the wear track experiences repeated contact separated by a non-friction period. Hiratsuka and Meki demonstrated that the non-friction time between successive sliding contacts affects metallic wear behavior because newly generated surfaces experience additional exposure to the surrounding atmosphere during the non-contact period, thereby modifying surface reactions and wear evolution [44]. In the present experiments, the interval between successive passages over the same track position was approximately 1.05 s at 0.03 m/s and 0.21 s at 0.3 m/s. Therefore, the longer non-friction interval under the low-speed condition provides additional time for environmental reactions of newly exposed surfaces and wear debris before subsequent contact, which is consistent with the formation of the thicker oxide-rich tribolayer observed at the lower speed.

The cumulative effect of repeated contacts and track geometry should also be considered when interpreting the tribolayer evolution. The low-speed condition involved approximately 9549 revolutions, compared with approximately 4775 revolutions at 0.3 m/s. Previous work on nanocrystalline Ni-W demonstrated that cumulative sliding history produces progressive changes in wear behavior and subsurface microstructure, highlighting the importance of repeated mechanical interactions during wear evolution [41]. Thus, the larger number of repeated contacts at 0.03 m/s provides more opportunities for material transfer, fragmentation, mechanical mixing, and reworking of the tribolayer. Furthermore, the different track radii used in this study modify the contact history by changing the track circumference, repeated interaction frequency, and debris circulation behavior. Although the independent influence of track geometry cannot be quantitatively separated in the present experiments, such configuration effects should be considered when interpreting third-body evolution and tribolayer development [45].

Overall, the distinct tribolayer architectures observed under the two sliding conditions should be considered as the integrated response to different sliding histories rather than the isolated effect of sliding velocity. The higher sliding speed reduces the duration of each sliding test and the number of repeated interactions experienced by a given position on the wear track, while simultaneously changing the deformation rate and contact conditions during sliding. In contrast, the lower sliding speed provides a longer exposure history, more repeated contact events, and a longer non-friction interval between successive contacts, which together promote surface oxidation, material transfer, and mechanical reworking of the third-body layer. Therefore, the thicker oxide-rich tribolayer formed at 0.03 m/s and the more developed metallic lamellar structure at 0.3 m/s represent the combined consequences of mechanically and environmentally coupled sliding conditions.

Interpretation of the wear-induced texture should account for the crystallographic state inherited from electrodeposition. Before sliding, the coating contained pronounced <1 1 1>//ND and <1 0 0>//ND fiber components in cross section; consequently, the texture measured beneath the wear track represents the extent to which frictional deformation modifies this pre-existing orientation distribution. The emergence of the B/$\bar{B}$ components at 0.30 m/s suggests that shear-related orientation selection becomes more prominent at the higher sliding speed. Severe plastic shearing beneath a sliding contact is known to generate characteristic texture components in FCC metals [13,26,46]. Which of these components becomes dominant is strongly influenced by the mechanisms responsible for accommodating the imposed strain. In coarse-grained Ni and other FCC metals with high stacking-fault energy, deformation is primarily carried by full dislocations, and the C component is commonly favored under simple shear [47]. Greater contributions from partial dislocations, stacking faults, or deformation twins, however, have been reported to promote the development of B/$\bar{B}$ components. This distinction is particularly relevant to nanocrystalline Ni because refinement to nanometer-sized grains can modify the relative contribution of different deformation mechanisms, including partial-dislocation activity and twinning processes [48]. In the present study, the HRTEM observations and corresponding FFT analysis (Figure 11a,b) revealed the presence of deformation twins within the highly deformed subsurface region. The observed twin boundaries and the corresponding matrix/twin crystallographic relationship indicate that twinning-related deformation occurred during sliding-induced plastic deformation. These results suggest that twinning may contribute to strain accommodation during sliding and may facilitate the development of B/$\bar{B}$ components. However, the formation of B/$\bar{B}$ components is interpreted primarily as a shear-related texture evolution rather than direct evidence of a single deformation mechanism. However, the contribution of individual deformation mechanisms cannot be quantitatively separated based on the present observations alone. At 0.03 m/s, deformation proceeded more gradually and produced less extensive crystallographic reorientation, leaving the initial <1 1 1>//ND and <1 0 0>//ND deposition textures comparatively well-preserved.

It should be noted that the present study has several limitations. The tribological behavior investigated here was limited to a single nanocrystalline Ni/AISI 52100 steel sliding pair and two representative dry-sliding conditions. In addition, the friction and wear measurements were obtained without independent statistical replication because of the limited availability of the nanocrystalline Ni coating specimens. Therefore, the observed differences should be interpreted as condition-specific responses associated with the investigated sliding conditions rather than generalized sliding-speed effects applicable to all Ni-based tribological systems. Further studies involving additional material combinations, repeated experiments using independent specimens, and broader sliding parameter ranges are required to establish more comprehensive relationships between sliding conditions, tribolayer evolution, and subsurface crystallographic reconstruction.

### 4.3. Strengths and Limitations of 4D-STEM for Tribolayer Characterization

Conventional TEM and SAED remain indispensable for phase identification and for establishing the overall stratified morphology of the tribolayer. However, in heavily deformed and nanocrystalline worn surfaces, these techniques necessarily average over regions that may contain multiple structural domains. Cai et al. noted that conventional TEM is invaluable for analyzing deformation structures in worn metals, but is less well-suited to quantifying texture in terms of its individual components [13]. More generally, orientation-mapping studies of nanostructured metals have emphasized that meaningful characterization of microstructural and textural gradients requires high spatial resolution and diffraction-based mapping approaches [49,50].

In the present study, 4D-STEM addresses this need by recording a diffraction pattern at every probe position [17]. This makes it possible to construct neighborhood correlation coefficient maps, to sum diffraction patterns over user-defined regions of interest, and to generate local orientation maps and ODF figures from the same dataset. These capabilities were crucial for revealing diffraction overlap, local misorientation, and friction-induced texture development within the tribolayer. In particular, the 4D-STEM analysis shows that the lamellar zone that formed at 0.3 m/s is a true shear-textured metallic layer, rather than only a morphologically flattened region.

At the same time, the interpretation of 4D-STEM data in nanocrystalline tribolayers must remain conservative. The method still projects a three-dimensional microstructure through a foil of finite thickness, and the diffraction pattern recorded at a single scan position may contain contributions from several overlapping grains. The general limitations of two-dimensional orientation mapping in nanostructured metals, including projection effects, indexing uncertainty, and the challenge of resolving very fine grains, have been emphasized in orientation-mapping studies and reviews [10]. These issues are particularly relevant here in the oxide-rich near-surface regions, where individual grains are only a few to tens of nanometers in size and overlap along the beam direction is frequent.

Accordingly, the strongest conclusions from the present 4D-STEM dataset are mesoscale rather than pixel-specific. The data robustly demonstrate the existence of diffraction overlap in the tribolayer, local lattice rotation or misorientation within some apparently grown grains, and the emergence of a simple-shear texture in the high-speed lamellar region. In contrast, unique orientation assignment at every individual scan position in grain overlapped regions should be interpreted with caution. Even with this limitation, 4D-STEM provides information that could not be obtained reliably from spatially averaged diffraction alone, and it substantially strengthens the mechanistic interpretation of sliding-speed-dependent tribolayer evolution in the present nanocrystalline Ni coating. Accordingly, future work using true three-dimensional orientation mapping in the TEM [51,52,53,54] should enable a more accurate visualization of worn microstructures in nanocrystalline metals by resolving projection overlap and capturing through-thickness orientation information.

## 5. Conclusions

The tribolayer architecture and subsurface crystallographic evolution of an electrodeposited nanocrystalline Ni coating sliding against AISI 52100 steel were characterized under dry sliding in air. For the present Ni/AISI 52100 steel tribosystem and testing conditions, the following conclusions can be drawn:

(1) Sliding speed strongly modifies the architecture of the evolved tribolayer. Low-speed sliding at 0.03 m s^−1^ forms a thick, porous, oxide-rich tribolayer, whereas high-speed sliding at 0.3 m s^−1^ produces a thinner oxide layer above a metallic lamellar subsurface region.
(2) The tribolayers are chemically mixed products of the Ni coating/AISI 52100 steel sliding pair. EDS analysis confirms oxygen enrichment and transferred Fe in the worn surface layers, indicating that both tribo-oxidation and counterbody transfer contribute to tribolayer formation.
(3) The two sliding speeds produce distinct surface and subsurface deformation modes. Low-speed sliding causes greater material pile-up and total topographic displacement, while high-speed sliding produces more pronounced valley formation and a more clearly developed metallic lamellar subsurface zone.
(4) 4D-STEM reveals sliding-condition-dependent local crystallographic evolution in the reliably indexed worn subsurface regions. The low-speed specimen contains a limited quasi-lamellar region with $A_{1}^{*}$/$A_{2}^{*}$ and C texture components, whereas the high-speed specimen develops a more pronounced shear-texture evolution involving C, B/$\bar{B}$, and $A_{1}^{*}$/$A_{2}^{*}$ components within the metallic lamellar zone. By comparing diffraction patterns acquired from adjacent probe positions and identifying grain boundary networks based on the corresponding orientation differences, 4D-STEM enables effective characterization of the wear-induced microstructural evolution in the near-surface region.

## Figures and Tables

**Figure 1:**
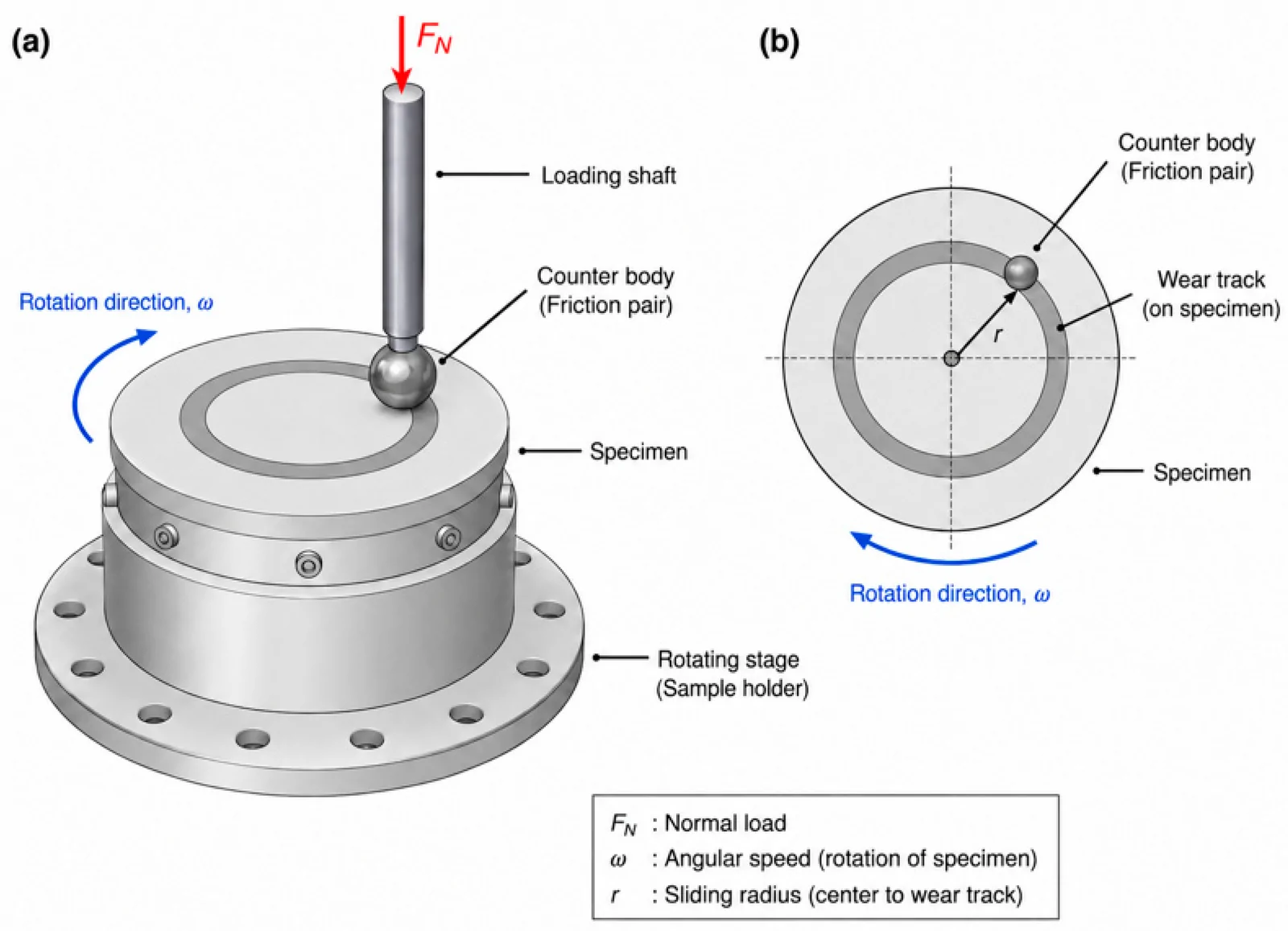
Schematic illustration of the ball-on-disk tribological test configuration used in this study: (**a**) three-dimensional view showing the counter body, loading shaft, specimen, and rotating stage; (**b**) top view illustrating the wear track geometry, sliding radius, and rotation direction. F_N_, ω, and r represent the normal load, angular velocity, and sliding radius, respectively.

**Figure 2:**
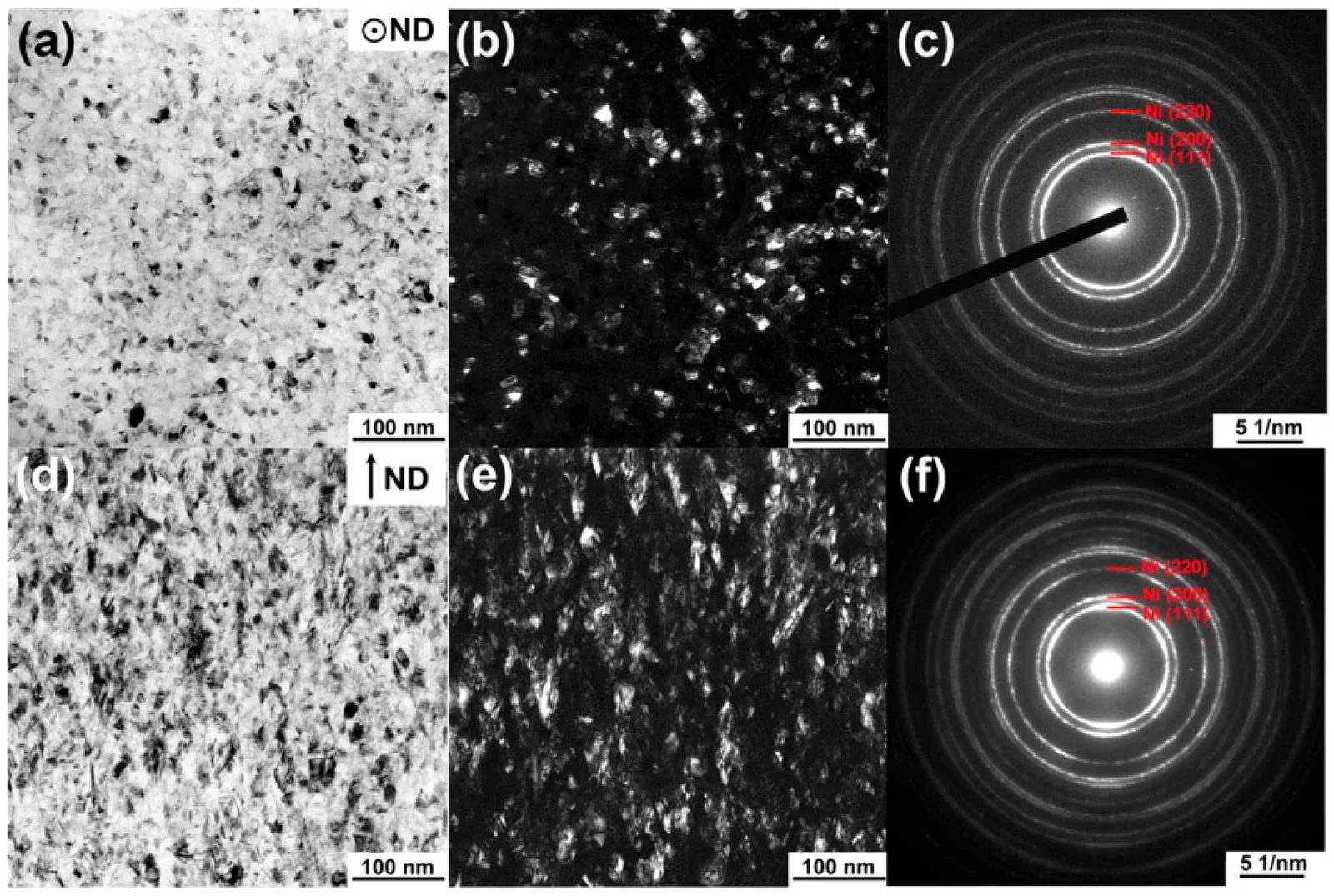
TEM characterization of the as-deposited nanocrystalline Ni coating: (**a**) plan-view bright-field image, (**b**) plan-view dark-field image, and (**c**) corresponding selected-area electron diffraction (SAED) pattern; (**d**) cross-sectional bright-field image, (**e**) cross-sectional dark-field image, and (**f**) corresponding SAED pattern. The {1 1 1}, {2 0 0}, and {2 2 0} diffraction rings are highlighted in (**c**,**f**).

**Figure 3:**
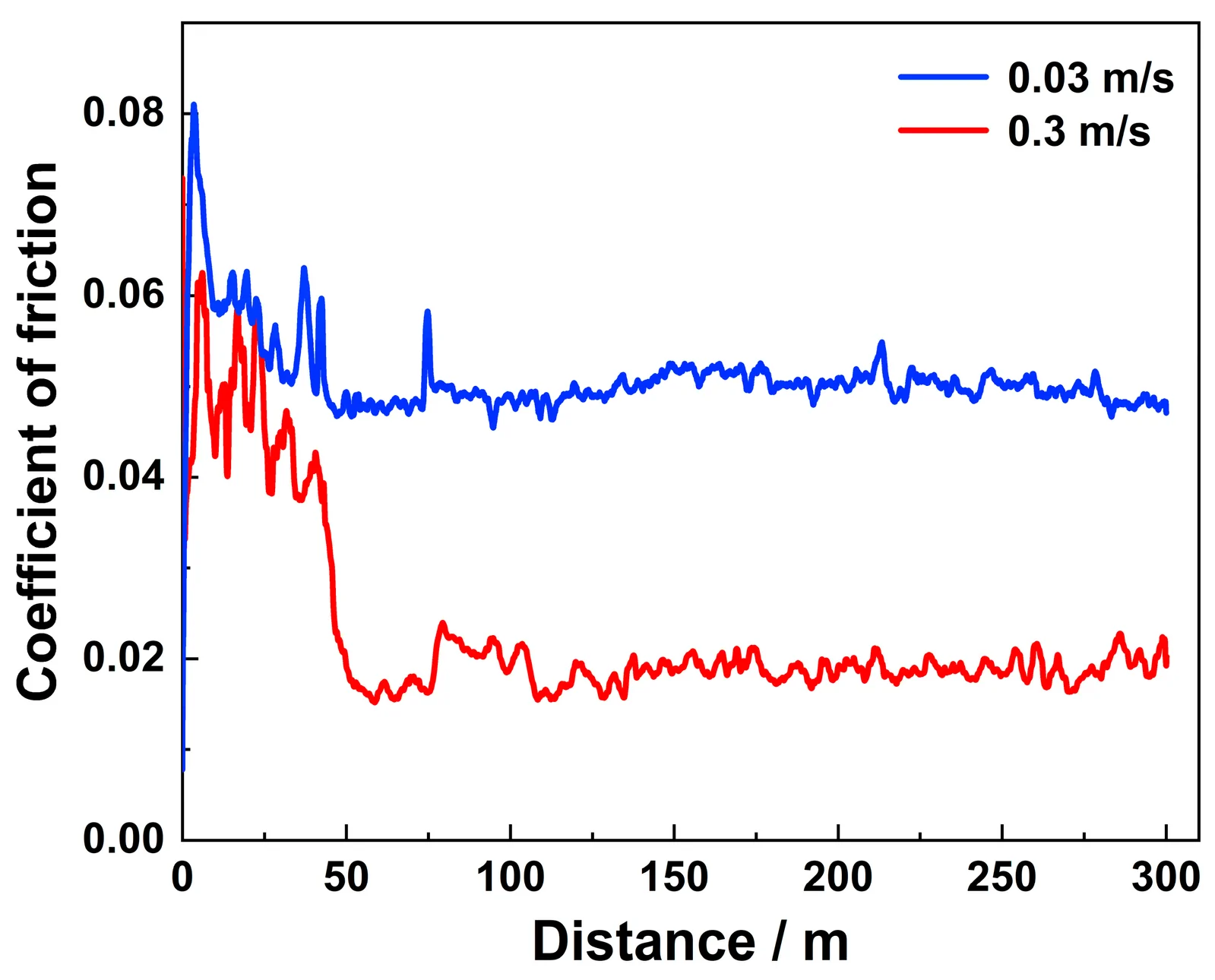
Coefficient of friction as a function of sliding distance for the nanocrystalline Ni coating tested at sliding speeds of 0.03 and 0.3 m/s.

**Figure 4:**
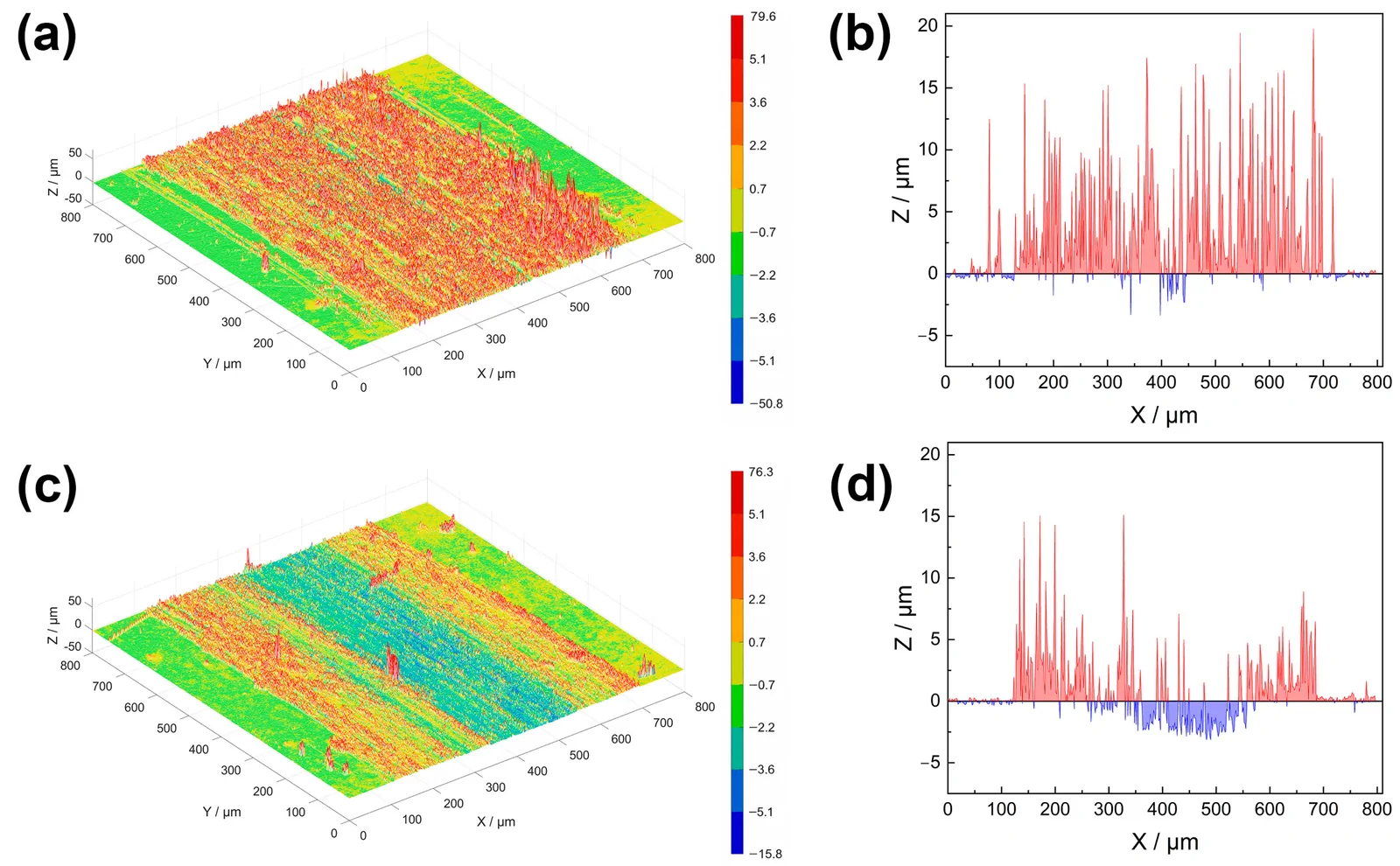
Three-dimensional confocal profiles of the wear tracks formed on the nanocrystalline Ni coating after sliding at (**a**) 0.03 and (**c**) 0.3 m/s; the corresponding two-dimensional cross-sectional profiles are shown in (**b**) and (**d**), respectively. Material below and above the reference surface is indicated in blue and red, respectively.

**Figure 5:**
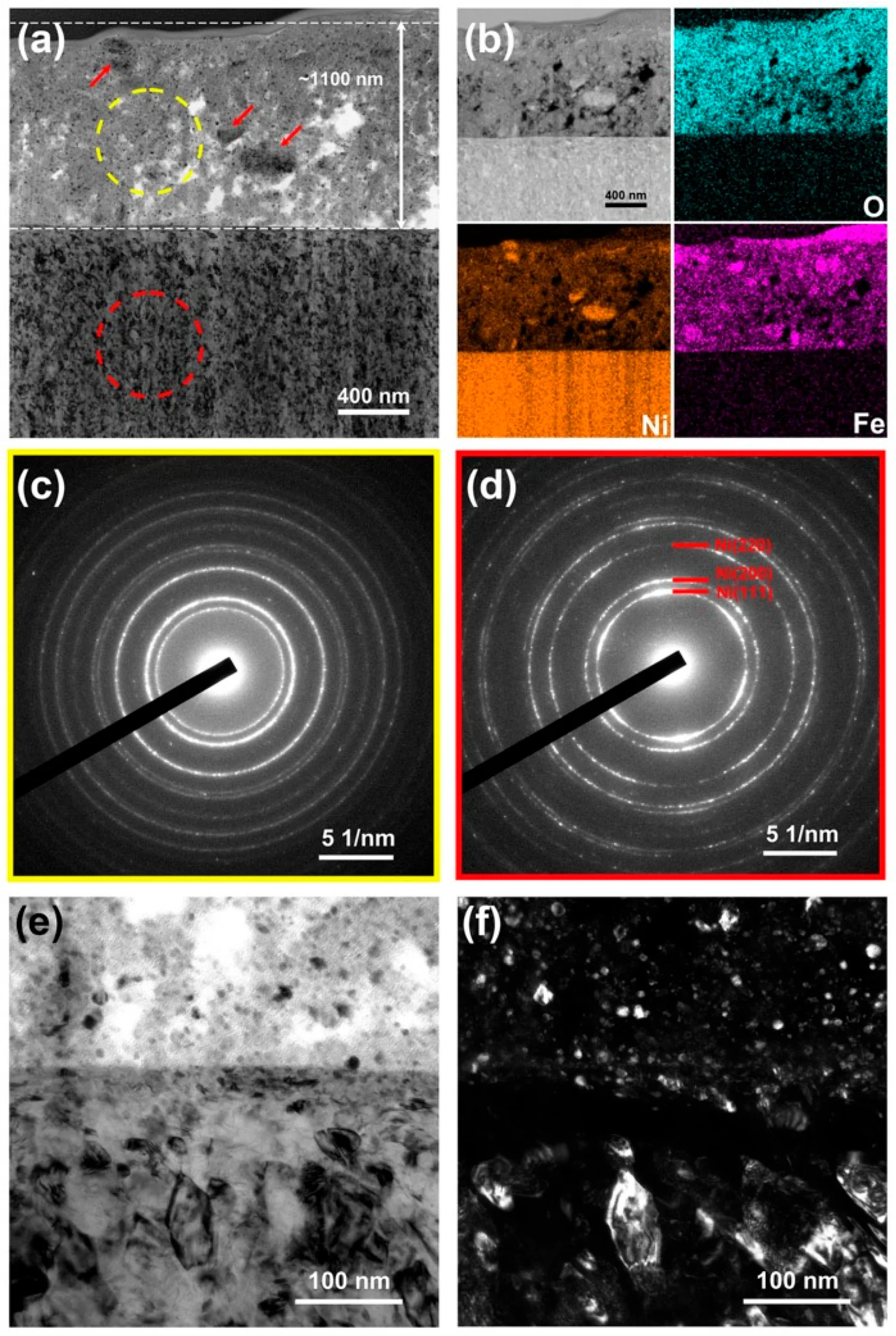
Cross-sectional TEM/STEM characterization of the tribolayer formed at 0.03 m/s: (**a**) bright-field TEM image, with red arrows indicating isolated Ni-rich domains, the white dashed line indicates the oxide-rich region with a thickness of approximately 1100 nm; (**b**) HAADF-STEM image with corresponding EDS elemental maps, where cyan, orange, and purple colors represent O, Ni, and Fe distributions, respectively; (**c**) diffraction pattern acquired from the tribolayer region marked by the yellow circle in (**a**), corresponding to the yellow frame; (**d**) diffraction pattern acquired from the subsurface region marked by the red circle in (**a**), corresponding to the red frame; (**e**) higher-magnification bright-field TEM image; (**f**) higher-magnification dark-field TEM image.

**Figure 6:**
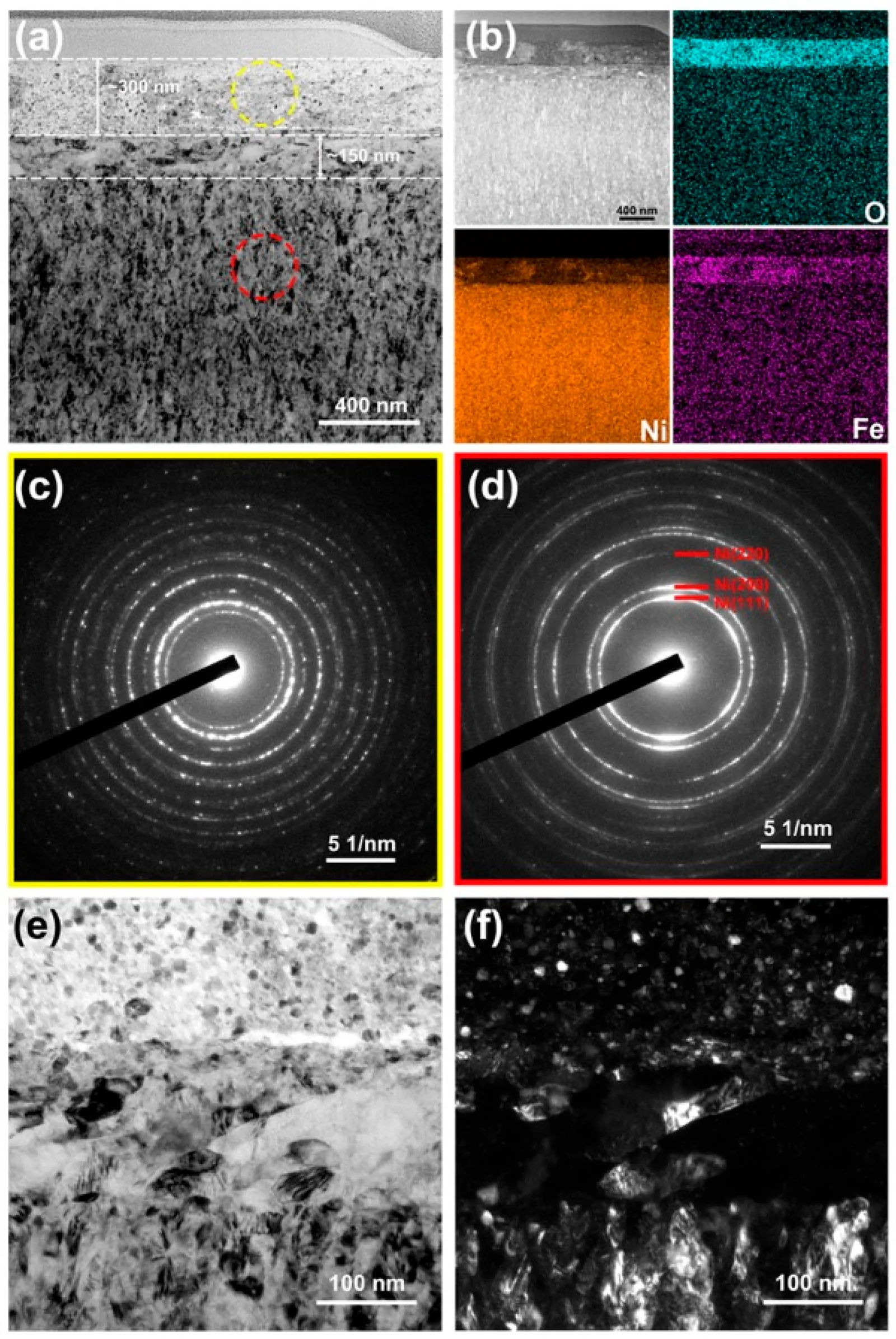
Cross-sectional TEM/STEM characterization of the tribolayer formed at 0.3 m/s: (**a**) bright-field TEM image, with the white dashed lines indicating the oxide-rich region with a thickness of approximately 300 nm and the grain coarsening region with a thickness of approximately 150 nm; (**b**) HAADF-STEM image with corresponding EDS elemental maps, where cyan, orange, and purple colors represent O, Ni, and Fe distributions, respectively; (**c**) diffraction pattern acquired from the tribolayer region marked by the yellow circle in (**a**), corresponding to the yellow frame; (**d**) diffraction pattern acquired from the subsurface region marked by the red circle in (**a**), corresponding to the red frame; (**e**) higher-magnification bright-field TEM image; (**f**) higher-magnification dark-field TEM image.

**Figure 7:**
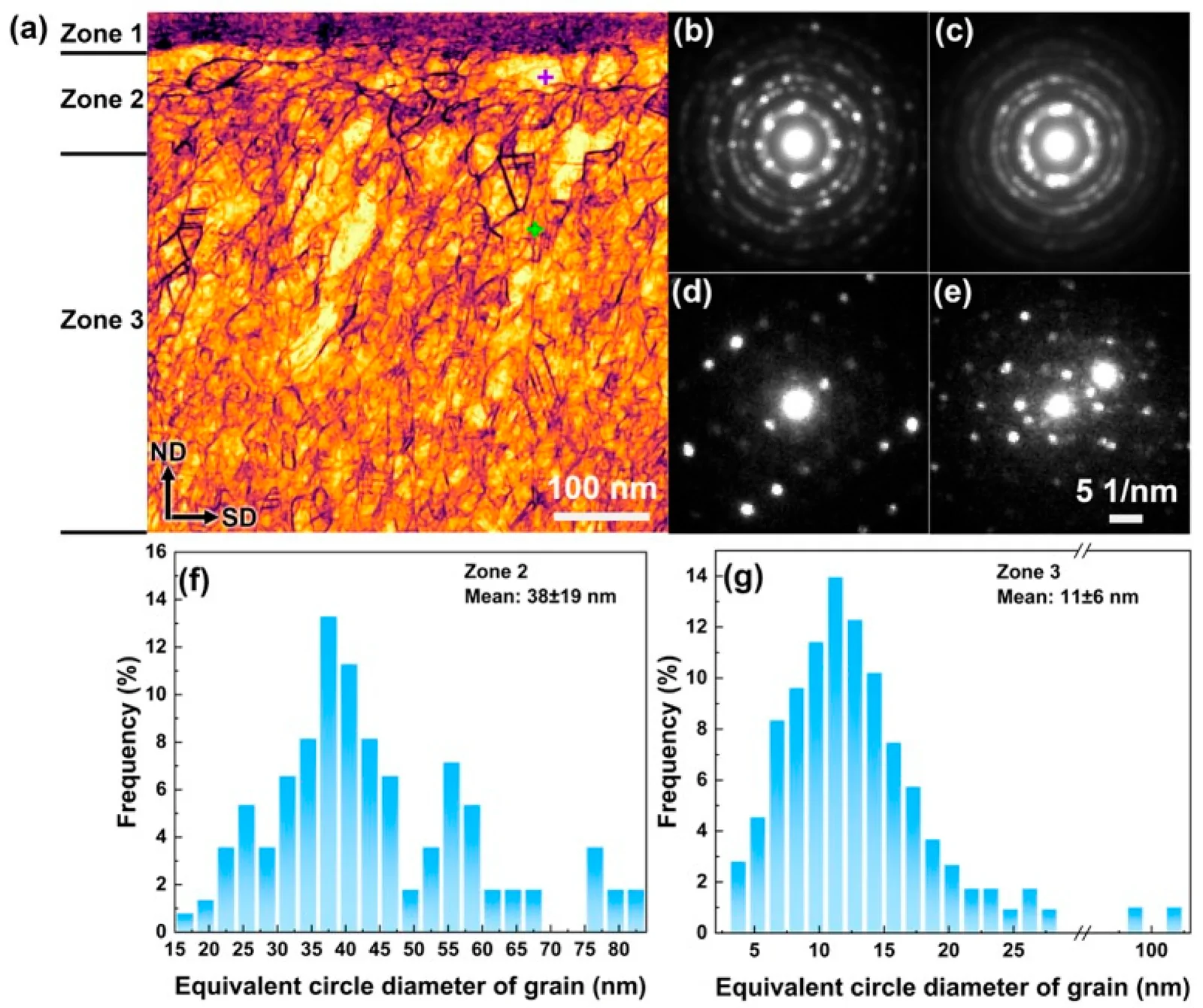
4D-STEM analysis of the specimen tested at 0.03 m/s: (**a**) correlation coefficient map with 512 × 512 pixels. Image intensity represents the local crystallographic continuity between neighboring pixels: bright regions indicate similar diffraction patterns, whereas dark regions reveal discontinuities associated with grain or phase boundaries, defects, or overlapping grains; (**b**) summed diffraction pattern from Zone 2; (**c**) summed diffraction pattern from Zone 3; and (**d**,**e**) representative diffraction patterns acquired from the regions marked by the purple and green crosses in (**a**), illustrating local diffraction variation and grain overlap. (**f**,**g**) Equivalent circle diameter distributions of grains in Zones 2 and 3, respectively. Grain boundaries for segmentation were identified by combining conventional dark-field TEM images, correlation coefficient maps, and virtual dark-field images. Grain sizes were calculated from the projected grain areas using the equivalent-circle diameter method. The distributions were obtained from 92 grains in Zone 2 and 178 grains in Zone 3.

**Figure 8:**
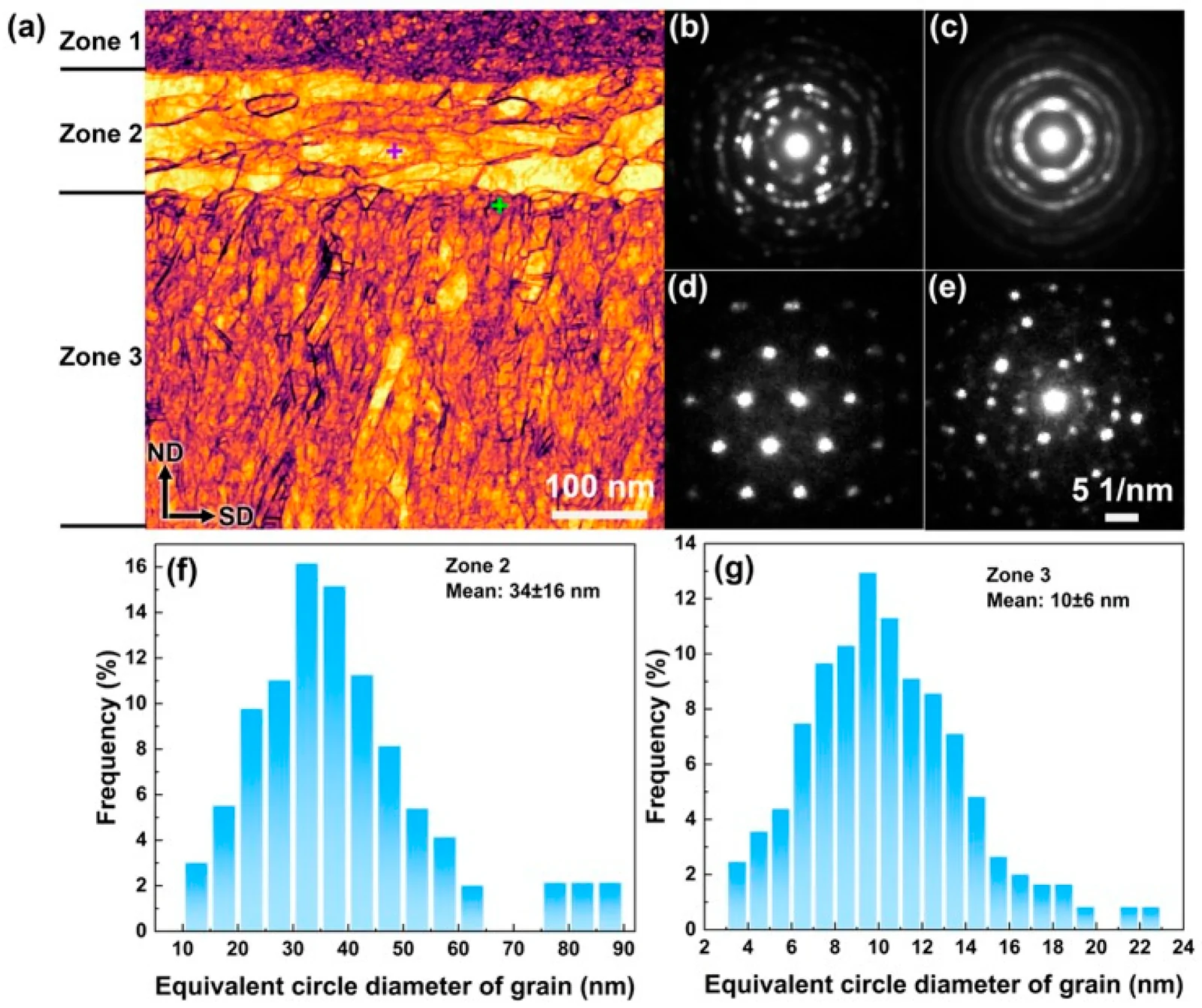
4D-STEM analysis of the specimen tested at 0.3 m/s: (**a**) correlation coefficient map with 512 × 512 pixels. Image intensity represents the local crystallographic continuity between neighboring pixels: bright regions indicate similar diffraction patterns, whereas dark regions reveal discontinuities associated with grain or phase boundaries, defects, or overlapping grains; (**b**) summed diffraction pattern from Zone 2; (**c**) summed diffraction pattern from Zone 3; and (**d**,**e**) representative diffraction patterns acquired from the regions marked by the purple and green crosses in (**a**), illustrating local diffraction variation and grain overlap. (**f**,**g**) Equivalent circle diameter distributions of grains in Zones 2 and 3, respectively. Grain boundaries for segmentation were identified by combining conventional dark-field TEM images, correlation coefficient maps, and virtual dark-field images. Grain sizes were calculated from the projected grain areas using the equivalent-circle diameter method. The distributions were obtained from 96 grains in Zone 2 and 166 grains in Zone 3.

**Figure 9:**
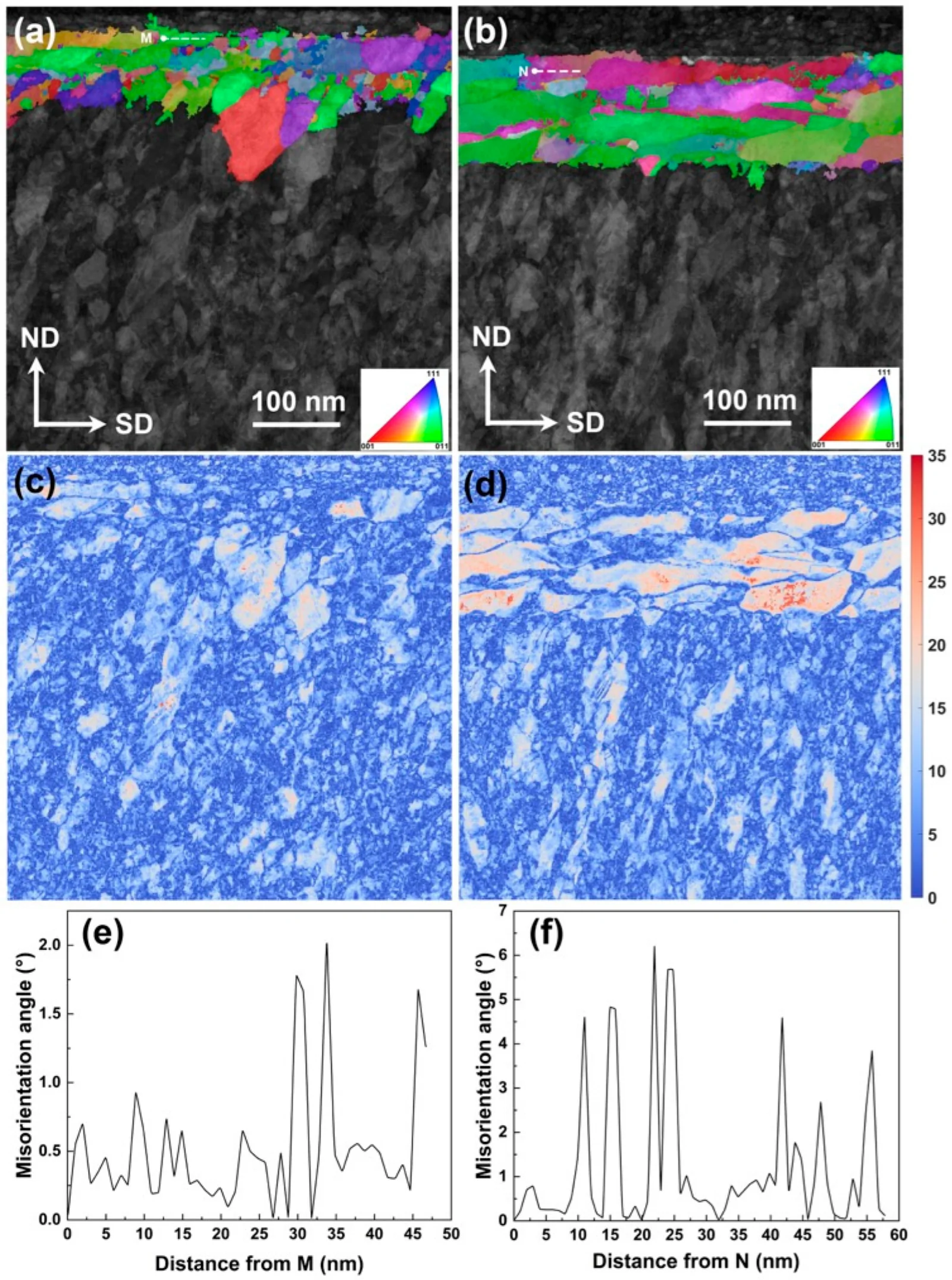
4D-STEM orientation analysis and indexing reliability evaluation of the near-surface regions in the specimens tested at different sliding speeds. (**a**,**b**) IPF orientation maps for the specimens tested at 0.03 and 0.3 m/s, respectively. Colors indicate the crystallographic direction parallel to the sliding direction, while the superimposed grayscale contrast reflects the confidence of local orientation indexing. (**c**,**d**) Corresponding orientation reliability maps for the IPF orientation maps shown in (**a**) and (**b**), respectively, illustrating the spatial distribution of indexing reliability. (**e**,**f**) Intragranular misorientation-angle profiles measured along the white dashed lines within representative grains in (**a**) and (**b**), respectively. The misorientation angle at each position along the profile was calculated relative to the crystallographic orientation at reference points M in (**a**) and N in (**b**).

**Figure 10:**
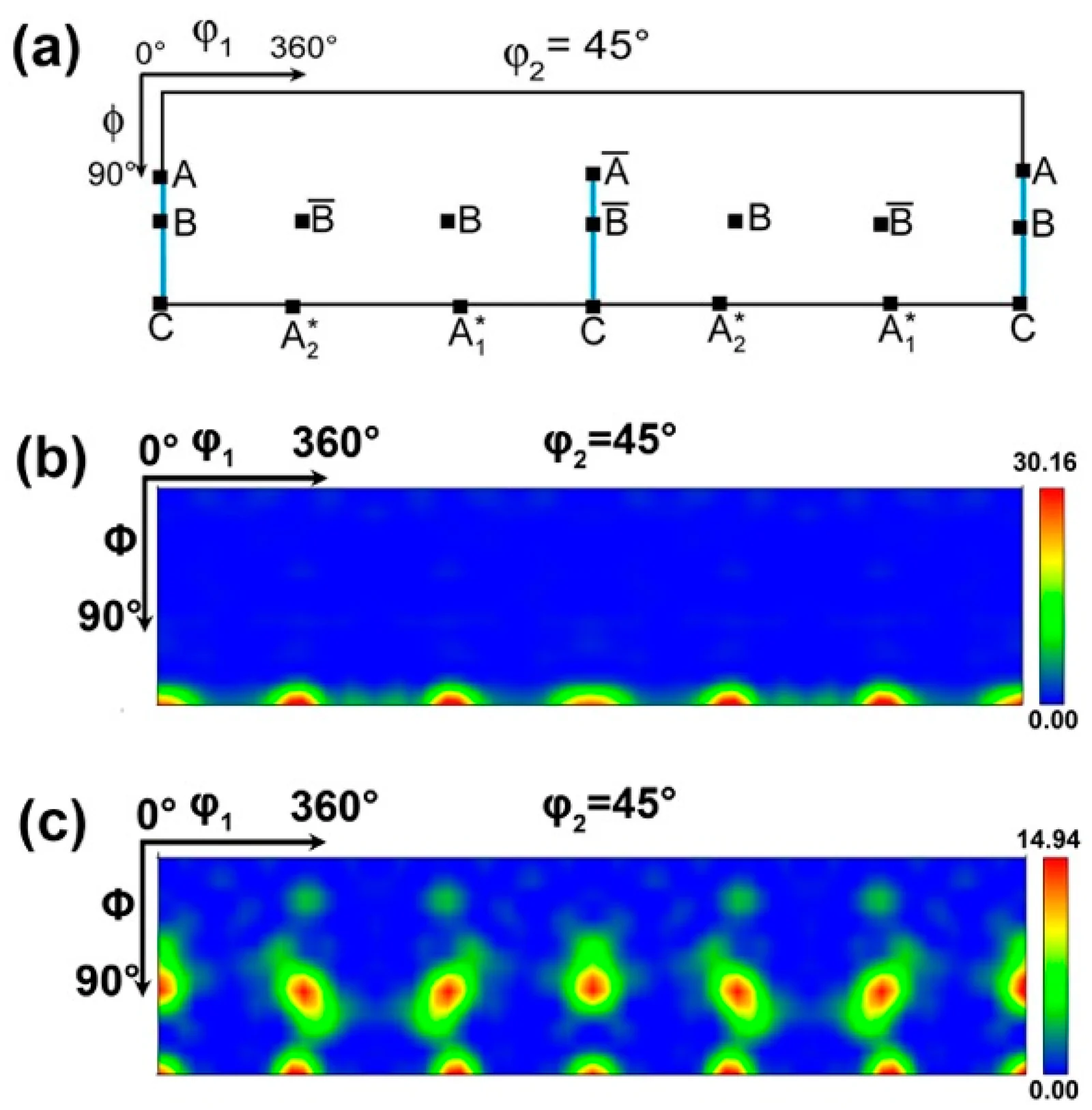
Crystallographic texture analysis derived from the 4D-STEM orientation data for the near-surface regions of the specimens tested at different sliding speeds. (**a**) Ideal FCC shear texture components in the φ_2_ = 45° ODF section [25]. ODF sections at φ_2_ = 45° for (**b**) the quasi-lamellar region in the specimen tested at 0.03 m/s and (**c**) the lamellar region in the specimen tested at 0.3 m/s.

**Figure 11:**
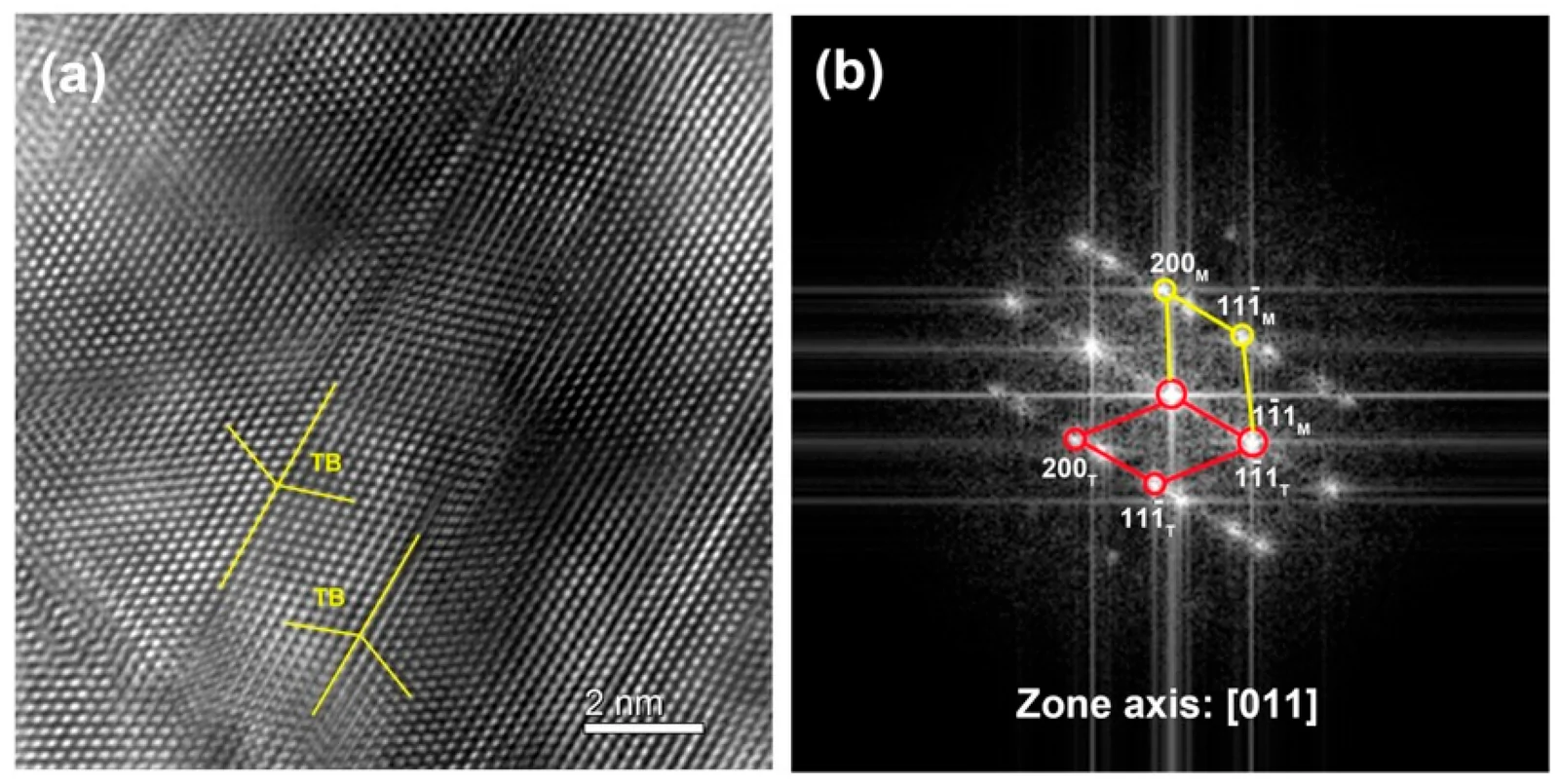
Evidence of deformation twinning in the high-speed sliding subsurface region: (**a**) high-resolution transmission electron microscopy (HRTEM) image showing twin boundaries (TBs) within the deformed lamellar structure; (**b**) corresponding fast Fourier transform (FFT) pattern indexed along the FCC Ni [011] zone axis, illustrating the crystallographic relationship between the matrix and twin regions.

**Table 1:** Experimental parameters for the ball-on-disk dry sliding wear tests.

Sliding Speed (m/s)	Distance from the Axis of Rotation Center (mm)	Sliding Distance (m)	Rotation Cycle	Sliding Time (s)
0.03	5	300	9549.3	10,000
0.30	10	300	4774.6	1000

**Table 2:** Volumetric parameters of the wear tracks measured by 3D confocal profilometry at different sliding speeds. The parameters include material removed below the reference surface, material piled up above the reference surface, and total displaced volume.

Sliding Speed (m/s)	0.03	0.3
Volume below reference surface (μm^3^)	6.01 × 10^4^	2.60 × 10^5^
Volume above reference surface (μm^3^)	1.97 × 10^6^	7.70 × 10^5^
Total displaced volume (μm^3^)	2.03 × 10^6^	1.03 × 10^6^

**Table 3:** Comparison of experimental d-spacings obtained from the tribolayer SAED patterns with reference values of the NiO, FeO, and metallic Ni phases.

Ring No.	Experimental d-Spacing (Å)	NiO	FeO	Ni
1	2.43	2.41	2.49	
2	2.08	2.09	2.16	2.03
3	1.74			1.76
4	1.48	1.48	1.52	
5	1.27	1.26	1.30	1.24
6	1.21	1.20	1.24	
7	1.06	1.04	1.08	1.06
8	0.98	0.96	0.99	1.02
9	0.94	0.93	0.96	

## Data Availability

The data presented in this study are available from the corresponding authors upon reasonable request. The data are not publicly available because they form part of an ongoing research project.
